# Endovascular stenting of traumatic extracranial carotid artery dissections in the pediatric population: a case report

**DOI:** 10.1186/1757-1626-2-171

**Published:** 2009-10-28

**Authors:** Joshua J Chern, Roukoz B Chamoun, Michel E Mawad, William E Whitehead, Daniel J Curry, Thomas G Luerssen, Andrew Jea

**Affiliations:** 1 Division of Pediatric Neurosurgery, Department of Neurosurgery, Texas Children's Hospital, Baylor College of Medicine, 6621 Fannin, Houston, Texas 77030, USA; 2 Division of Neuroendovascular, Department of Radiology, Baylor College of Medicine, 6621 Fannin, Houston, Texas 77030, USA

## Abstract

**Background:**

Traumatic extracranial carotid artery injuries are rare in children. Treatment options include surgery, anticoagulation therapy and endovascular treatment. There have been only limited reports in the literature documenting endovascular carotid stent placement in the pediatric population. In these reports, stent deployment was utilized in the setting of pseudoaneurysm formation.

**Case report:**

Here we report a case of endovascular carotid stenting for traumatic carotid dissection in a 15 year-old boy. An uncovered bare self-expanding stent was used to treat progressive high-grade stenosis in the early post-injury period.

**Conclusion:**

At 6 months post-procedure, complete healing of the carotid artery was demonstrated on follow-up angiogram.

## Case report

### History & Physical Exam

A 15-year-old previously healthy boy was elbowed in the neck during a football game. He continued to play after the incident; however, later on that day, he started having headache which prompted his visit to the Emergency Room. He had no other symptoms, and his neurologic exam was normal.

### Initial work-up

**Figure 1:**
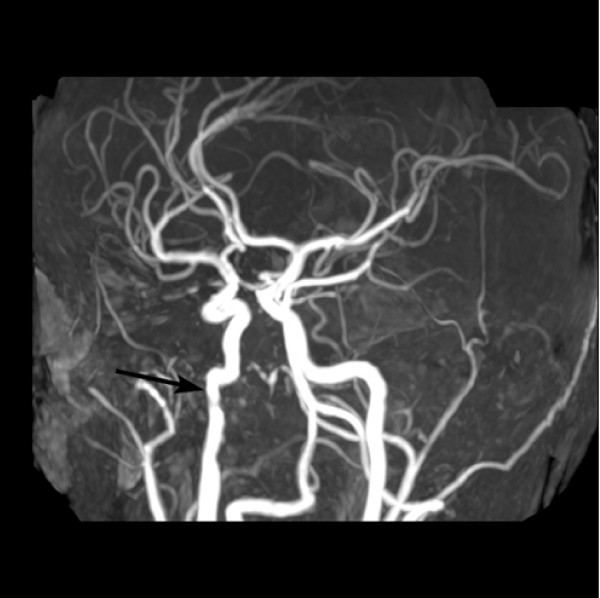
**3D reconstructed time-of-flight MRA shows very minimal irregularity of the distal right ICA just below the skull base consistent with a small focal dissection at this site (arrow)**.

A CT scan of the head was done, and it was suspicious for a right internal carotid artery (ICA) dissection at the level of the skull base involving the extracranial portion of the artery. MRI and MRA of the brain and neck were subsequently performed and confirmed the diagnosis of a dissection resulting in minimal narrowing of the lumen of the ICA (Fig 1). The patient was admitted to the hospital for observation, and was started on aspirin 81 mg daily. Work-up for collagen vascular disorders which may have predisposed him to carotid dissection was negative. He remained neurologically and was discharged home.

### Follow-up Imaging

**Figure 2:**
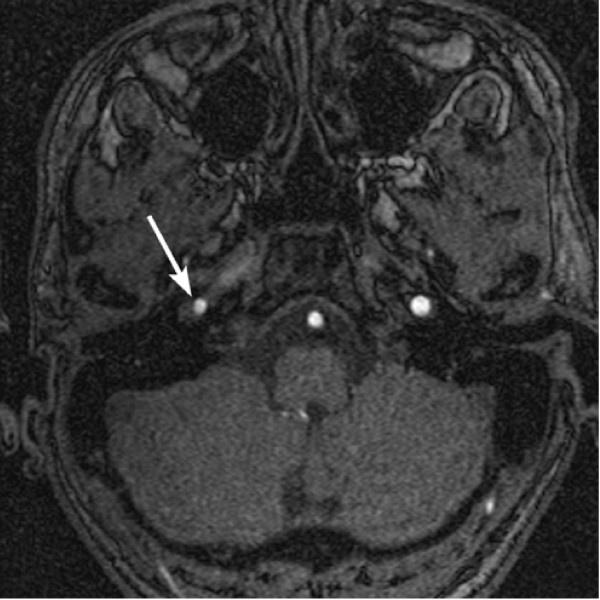
**Axial source images of 2D time-of-flight MRA at 1-month postinjury shows an intimal flap (arrow) within the right ICA at the skull base extending for approximately 1.7 cm**. The true lumen of the vessel is compressed by the false lumen.

**Figure 3:**
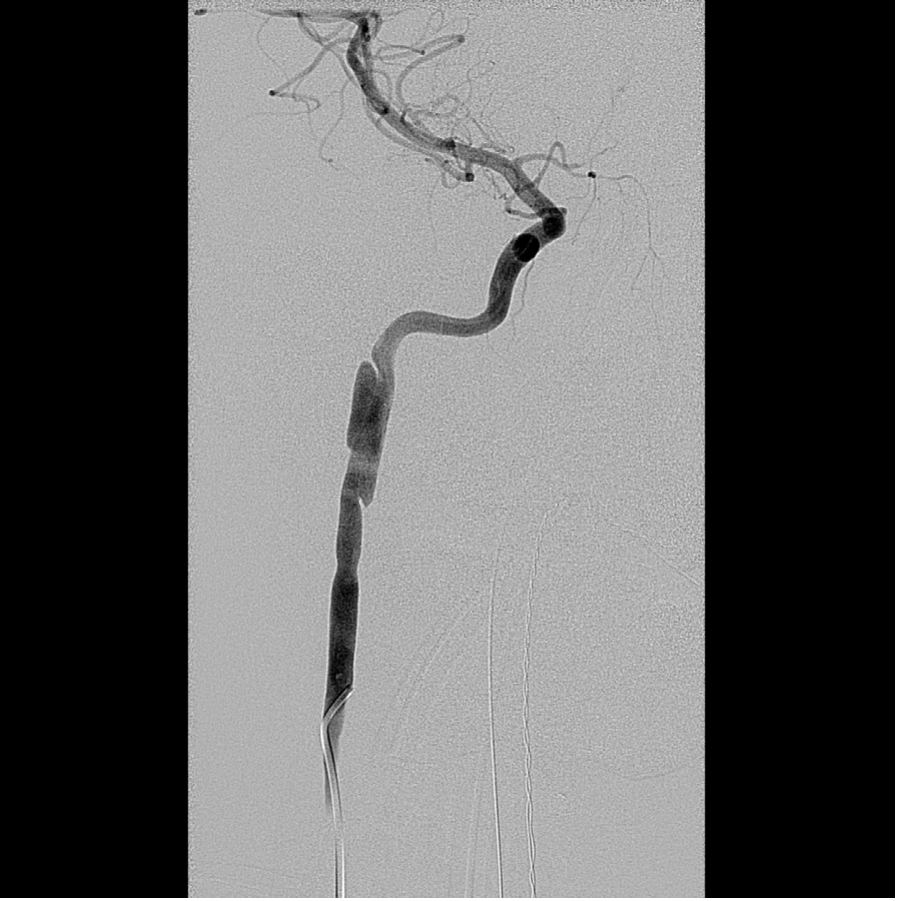
**Conventional angiography with right cervical ICA injection confirms a spiral dissection with high-grade stenosis of the true lumen**.

At 1 month of follow-up, he was asymptomatic with significant improvement of his headache. Follow-up MRI and MRA were performed and showed an unexpected progression of the dissection with high-grade stenosis of the true lumen of the right internal carotid artery (Fig 2). The finding was confirmed with conventional angiography. Clopidogrel was added in addition to aspirin. After consulting the patient and his family, the patient underwent stenting of the right ICA at 2-month postinjury (Fig 3).

### Procedure

Using a micropuncture set and under fluoroscopic guidance and strict sterile technique a 6 French femoral sheath was inserted into the right common femoral artery. ACT was kept between 250 and 300 with systemic anticoagulation.

The pretreatment internal carotid arteriogram demonstrated a persistent spiral dissection of the distal cervical segment of the right internal carotid artery with associated pseudoaneurysm. Following the diagnostic angiogram, and via a right internal carotid artery a small microcatheter was advanced over the wire through the spiral dissection. Over an exchange wire a self-expanding nitinol stent (Precise/Cordis), which measured 5 × 40 mm, was successfully deployed in the cervical segment of the left internal carotid artery across the spiral dissection.

Follow-up arteriogram shows good apposition of the stent to the arterial wall with restoration of the luminal diameter with diminished filling of the aneurysm and stagnation of the contrast within the sac of the aneurysm. There is no evidence of platelet aggregation, or major branch occlusion.

### Post-procedural course

**Figure 4:**
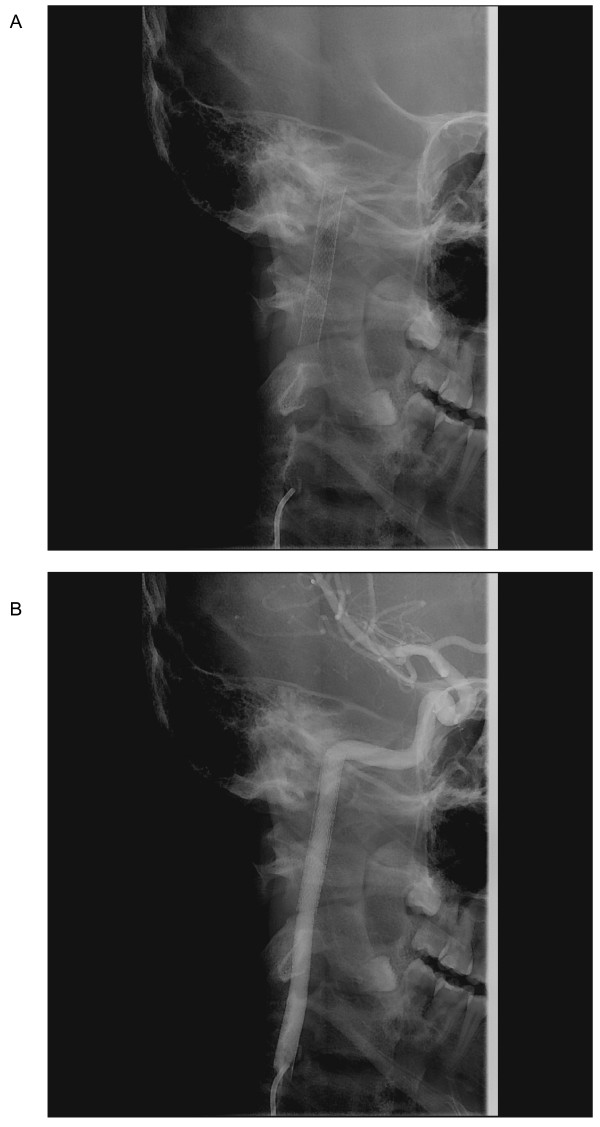
**(A) Oblique neck xray shows stent deployment, and (B) follow-up catheter angiography at 6-months poststenting with right cervical ICA injection shows restoration of the normal carotid lumen without vessel wall**.

At 6-month post-stenting, the patient remained asymptomatic. Angiogram showed complete resolution of the dissection without residual stenosis and with evidence of endothelialization of the stent (Fig 4). Clopidogrel was stopped, and he was maintained on aspirin. The patient will be followed clinically and with annual CT angiogram. We would also stop aspirin if the result of CT angiogram is satisfactory without wall abnormalities at one year post-stenting.

## Discussion

The incidence of carotid dissection in adults after blunt head and neck injury is estimated at 0.3 to 0.67% [1,2]. In children, this injury seems to be significantly less common (estimated at 0.03%) [3]. However, because carotid artery dissection can be clinically silent, its frequency may be underestimated.

Arterial dissection has been associated with a number of conditions including fibromuscular dysplasia, Marfan syndrome, cystic medial necrosis, oral contraceptives, and drug abuse. In these cases, there is a structural defect in the arterial wall predisposing to dissection either spontaneously or after a minor trauma. On the other hand, traumatic dissection is also known to occur in otherwise healthy patients. As a result, the pathophysiology, natural history, and prognosis are different for traumatic versus spontaneous dissection.

It has also been reported that intracranial and extracranial dissection can have different clinical presentations. Intracranial dissection can predispose to subarachnoid hemorrhage. Extracranial dissection most commonly present with ischemic signs and symptoms. Spontaneous carotid dissections are more commonly intracranial, while traumatic ones are more commonly extracranial.

### Management options

The natural history of asymptomatic carotid dissection remains unknown; therefore, the treatment for asymptomatic traumatic extracranial carotid dissection is controversial.

First-line medical therapy is based on the premise that the majority of neurologic events are related to thrombus within the lumen and are potentially preventable with anticoagulation or antiplatelet drugs [4-6]. Surgical intervention for carotid dissection is reserved for patients with recurrent TIAs or progressive neurological deficits secondary to hypoperfusion or embolic phenomenon despite maximal medical therapy. One study indicated that the presence of a large or expanding pseudoaneurysm, occurring in one-third of cases, is also an indication for surgical intervention [7]. Chronic carotid dissections have also been treated with surgical reconstruction to prevent further ischemic or thromboembolic complications, if medical treatment with six month anticoagulation fails or if carotid aneurysms and/or high grade stenosis persist [8].

In most cases, endovascular treatment has supplanted open surgery as the initial treatment of choice once medical therapy fails in adults [9,10]. However, experience in endovascular carotid stenting in children is much more limited, and long-term results of carotid stenting in children are unknown, and the treatment of stent-related complications can be complex [11].

### Endovascular Stenting in Children

Only 9 cases of endovascular stenting in the pediatric population, including our case, have been reported for extracranial carotid artery injury. 7 of these cases were trauma-related, and the other two cases were idiopathic. The indication for stenting in all these cases was pseudoaneurysm formation. In our case, endovascular stenting was been performed for an unexpected worsening of the carotid dissection and progressive carotid stenosis in the subacute radiographic follow-up period.

Signs and symptoms in these patients included pain, local neck swelling and ischemic symptoms. However, 4 of these patients were neurological intact at time of diagnosis. Despite the common belief that conservative anticoagulant therapy is the first line of treatment, only one patient underwent anticoagulant therapy at the time of diagnosis. This would imply a more aggressive approach, but this is more likely a result of reporting bias. The complication rate of the treatment was significant. In 3 cases additional endovascular treatment was needed to treat recurrent/residual pseudoaneurysm; no complications occurred in the peri-procedural period. The follow-up for these cases ranged from 3 - 25 months. There have been no reports documenting long-term follow-up in these patients. The incidence and rate of progressive carotid stenosis after stenting is of particular concern and interest over the lifetime of a child.

Endovascular stent treatment in the pediatric population raises technical concerns over vessel caliber - both carotid artery and femoral artery. In a child, the femoral artery may be too small to accommodate the introduction of a sheath and shuttle endangering its patency. However, the decreased tortuosity of pediatic vessels makes stent placement feasible in the extracranial carotid artery [12].

## Consent

Written informed consent was obtained from the patient for publication of this case report and accompanying images. A copy of the written consent is available for review from the journal's Editor-in-Chief.

## Competing interests

The authors declare that they have no competing interests.

## Authors' contributions

JJC was responsible for the concept and design of the manuscript and for writing and/or editing of the manuscript. RBC aided in the editing of the manuscript. MEM analyzed and interpreted the radiographic data for this case. WEW aided in the editing of the manuscript. DJC aided in the editing of the manuscript. TGL aided in the editing of the manuscript. AI analyzed and interpreted the radiographic data related to the case. AJ was responsible for the concept and design of the manuscript and for writing and/or editing the manuscript. All authors read and approved the final manuscript.
